# 

**Published:** 1892-06

**Authors:** 


### Kentucky State Dental Association.

The Twenty-Second Annual Meeting of the Kentucky State Dental Association will be held at the Louisville College of Dentistry, Louisville, Ky., commencing Tuesday, June 21st, 1892, and continuing to the 23d.

The following programme has been prepared:

PAPERS.

"The Care of Children's Teeth," by Dr. S. T. Butler, Leitchfield, Ky. Discussion to be opened by Dr. J. B. Alexander, Louisville, Ky.

"Effects of Acquirement Upon Heredity," by Dr. A. O. Rawls, Lexington, Ky. Discussion to be opened by Dr. J. S. Cassidy, Covington, Ky.

"Dental Caries," by Dr. M. W. Steen, Augusta, Ky. Discussion to be opened by Dr. Wm. Van Antwerp, Mt. Sterling, Ky.

"Antiseptics," by Dr. J. S. Cassidy, Covington, Ky. Discussion to be opened by Dr. J. C. Blair, Louisville, Ky.

"Defects of Palate," by Dr. G. Molyneaux, Cincinnati, Ohio. Discussion to be opened by Dr. H. B. Tileston, Louisville, Ky.

"Gold Crowns and Bridge Work," by Dr. C. G. Edwards, Louisville, Ky. Discussion to be opened by Dr. B. Oscar Doyle, Louisville, Ky.

"Educating the Public," by Dr. Henry Pirtle, Louisville, Ky. Discussion to be opened by Dr. B. Oscar Doyle, Louisville, Ky.

A paper by Dr. J. F. Rees, Owenton, Ky. Subject to be announced.

CLINICS.

All Clinics will be given in the Infirmary of the Dental College, at such hours as may be announced.

The State Board of Examiners will meet daily during the session to examine and register applicants.

---

The Willard Hotel has been selected as head-quarters, and a special rate arranged for; there will also be a reduction in railroad fares.

A cordial invitation is extended to all Members of the Dental Profession.

Members or visitors having anything new in appliances, or methods, will have proper time allotted to them by applying to the Executive Committee.

Dr. J. H. Baldwin, Secretary,
Louisville, Ky.

### Hymeneal.

MARRIED on Saturday, May 14, 1892, Dr. Alice Sherman, D.D.S., to Mr. Joel Barber, both of Lake Geneva, Wis.

Congratulations are now in order, not only from the friends and acquaintances of their home and vicinity, but from a host of friends of the bride made during her residence in the University of Michigan.

We will venture to speak for them till she can hear from each personally.

We congratulate each for having the best companion in the world.

We will suggest to Mr. Barber that he may find it necessary to submit to a division of his good wife's affection, for she did love her profession, and doubtless does yet, as we are informed she will continue her dental practice at her former office.

May their pathway through life grow brighter and brighter till they arrive at the home of perfect bliss.

### CONTENTS.

### COMMUNICATIONS.

### THE DENTAL REGISTER,

**A MONTHLY MAGAZINE DEVOTED TO THE INTERESTS OF THE**

### DENTAL PROFESSION.

*Terms Reduced to $2.00 Per Year. Single Copies, 25 Cents.*

**EDITED BY PROF. J. TAFT, D.D.S.**

—AND—

**Published by SAM'L A. CROCKER & CO., Ohio Dental and Surgical Depot,**

The volume commences in January and closes in December.

The REGISTER being issued monthly is always fresh, therefore Indispensable to the practitioner who desires to keep posted up with the new inventions and improvements which are daily developed. Neither expense nor effort will be spared to give value and variety to its contents. Articles will be illustrated with well executed engravings whenever they will add to the interest or assist to explanation.

Its extensive circulation and frequency of issue make it one of the BEST DENTAL ADVERTISING MEDIUMS in the United States.

All subscriptions must begin with January or July.

Local or special notices, five lines or less, will be inserted for $3 each insertion; over five lines, 50 cts. per line.

All advertising bills payable in advance, or at furthest, after the issue of the first number containing the advertisement. All transient advertisements to be paid for in advance.

☞ CONTRIBUTIONS SOLICITED.

Exclusively Editorial Communications should be addressed to the Editor.

The latest number of the REGISTER may be ordered with or without other goods at any time, and all letters should be addressed to

SAM'L A. CROCKER & CO., Ohio Dental and Surgical Depot, Cincinnati, O.

